# Comparative effectiveness of school-based caries prevention: a prospective cohort study

**DOI:** 10.1186/s12903-018-0514-6

**Published:** 2018-03-27

**Authors:** Ryan Richard Ruff, Richard Niederman

**Affiliations:** Department of Epidemiology & Health Promotion, New York University College of Dentistry, 433 First Avenue, New York, NY 10010 USA

**Keywords:** Oral health, Caries prevention, Comparative effectiveness

## Abstract

**Background:**

Dental caries is the world’s most prevalent childhood disease. School-based caries prevention can reduce the risk of childhood caries by increasing access to care. However, the optimal mix of treatment services, intensity, and frequency of care is unknown.

**Methods:**

Data were derived from two prospective cohorts of US children participating in two caries prevention programs with different treatment intensities. One program provided primary and secondary prevention (glass ionomer sealants and interim therapeutic restorations) and one primary prevention only (glass ionomer sealants), both given twice yearly in six-month intervals. Primary study outcomes included untreated decay and the total observed caries experience. Analysis used generalized additive models to estimate nonlinear effects and trends over time. Results were compared to those estimated using generalized estimating equations and mixed-effects multilevel Poisson regression.

**Results:**

Primary and secondary prevention combined did not significantly reduce total caries experience compared to primary prevention alone, but did reduce the risk of untreated decay on permanent dentition. Additionally, the rate of new caries experience was slower in the primary and secondary prevention group. Nonlinear trends for dental caries across both programs were statistically significant from zero (*p* < .001).

**Conclusion:**

Caries prevention consisting of primary and secondary prevention agents may be more effective than primary prevention alone in reducing the risk of tooth decay over time. Results suggest that the impact of caries prevention may not be constant over the medium- and long-term, suggesting reduced effectiveness with continued treatments.

## Background

Dental caries is the world’s most prevalent childhood disease [1], accounts for 3.5 million disability-adjusted life years [2], and affects nearly 30% of school-age children and 50% of rural, minority, or Medicaid recipient children in the United States [3, 4]. Caries prevention programs such as one-chair services, mobile dental vans, and school-based health programs or dental centers can be used to reduce the risk of tooth decay [5]. These school-based programs improve access to dental services in medically underserved areas, and often serve as the sole source of dental care for children [5]. Based on evidence from multiple systematic reviews, the American Dental Association supports the use of school-based programs that apply dental sealants to sound surfaces and noncavitated lesions to reduce dental caries in children [6, 7].

The comparative effectiveness of caries prevention delivery models has been identified as one of the highest research priorities by the Institute of Medicine [8]. Importantly, there is considerable variability both within and across caries prevention programs, such as the types of preventive services that are provided and the timing, frequency, and intensity of care. Thus, questions remain as to the effectiveness of caries prevention, such as how much prevention is needed, with what frequency, or whether secondary prevention methods (e.g., interim therapeutic restorations, silver diamine fluoride) are more effective when combined with primary prevention (e.g., varnish and sealants), compared to primary prevention alone. Additionally, research on the long-term impact (e.g., at least one or two years of follow-up) of school-based caries prevention programs is largely limited to prevention consisting of fluoride or sealants [6, 9–11]. Further research is therefore needed to identify the optimal mix of services and implementation strategies that can be used to effectively treat childhood caries.

In this study, we compared two caries prevention programs with identical frequency of care but different treatment agents. We explored the long term, nonlinear trends in untreated decay and total caries experience within program over time. We hypothesized that increased intensity of care, defined as primary and secondary prevention combined, would result in greater reduction in untreated tooth decay and that effects across both programs would be nonlinear over time. Results from this research can begin to inform best practices in program design and implementation and lead to a more robust, effective caries prevention program.

## Methods

### Design and population

Data were derived from two prospective open cohorts of primary school-aged children receiving school-based caries prevention in urban and rural elementary schools in the northwestern and central United States from 2004 to 2014. These populations were chosen as they are characterized by low-income children who typically lack adequate access to preventive dental care. Many of the participating children did not have community water fluoridation. All participating schools were designated as Title 1, as over half of enrolled students qualified to receive reduced price meals. All children were eligible for the study regardless of age, gender, race, or socioeconomic status.

In each year of the program, students in participating schools were provided informed consent. Children who had completed informed consent were followed longitudinally and treated twice yearly with caries prevention for as long as they were enrolled in the school. Children in the northwestern US cohort received primary and secondary prevention, and children in the central US cohort received primary prevention only. Visits for each participant were numbered sequentially regardless of the time that had elapsed between visits. Subjects were included in analysis only if they were between the ages of 5 and 12. Overall, there were 46 participating schools.

### Interventions

The first cohort (“unexposed”) included participants from a traditional school-based sealant program. The second cohort (“exposed”) received both primary and secondary prevention. Participants in the unexposed group received a twice-yearly dental evaluation conducted by a dental hygienist, oral hygiene instruction (mirror, toothbrush, toothpaste, and floss), prophy, fluoride varnish, and glass ionomer sealants placed on permanent first and second molars. Participants in the exposed group received all preventive interventions given to controls with the addition of sealants placed on all teeth and glass ionomer interim therapeutic restorations (ITRs) placed on all asymptomatic teeth with carious lesions.

### Data collection, calibration, and standardization

At each observation for each participant, examining dentists dried tooth surfaces with gauze squares and performed clinical visual-tactile full-mouth oral examinations, including examination of all teeth and tooth surfaces for decayed, missing, filled, sound, or pulpal involvement. The exam also included an assessment of pain, swelling, infection, and abscess. Dental hygienists delivered all services with the exception of clinical oral exams, which were given by a licensed dentist. Each examiner recorded clinical data using a proprietary tablet-based software which was then securely uploaded to a Data Coordinating Center for cleaning and quality control.

For calibration, data collectors examined ten students independently at baseline and discussed whether caries were present or not. Following this review, data collectors were calibrated by examining another ten students independently and comparing results (kappa = 0.75). To standardized delivery of care, hygienists were trained to use Fuji IX glass ionomer in capsules prior to participating in the program. Following the start of the program, dentists and hygienists were standardized each year but not calibrated.

### Outcomes

Outcome measures included the prevalence of untreated decay on permanent dentition and the total observed caries experience (TOCE). Untreated decay was calculated as any permanent tooth or tooth surface with any untreated carious lesion and analyzed at the person-level. TOCE was calculated as the sum of all decayed or filled teeth observed over the course of the study, regardless of exfoliation. For example, if a child presented at the first follow-up visit with a cavity on a single tooth, their TOCE score would be one. If at the second follow-up visit, the child presented with a different tooth having a cavity, and the previously observed tooth is either cavitated, missing, or filled, their TOCE score would be two. Previously observed teeth with decay or fillings that were later exfoliated are carried forward and contribute to the overall TOCE score. In this way, TOCE reflects the total number of teeth ever observed with decay over the course of the study. TOCE is useful in the analysis of longitudinal caries prevention data, and is preferred to more common measures, as the TOCE score does not decrease due to tooth exfoliation [12]. Other tooth indices are potentially biased due to decreases over time from the exfoliation of previously decayed teeth, a decrease that would be mistakenly attributed as a treatment effect.

### Statistical analysis

Primary analysis used generalized additive models (GAMs) to assess decay prevalence and TOCE scores. Notably, there is little research on the long-term effects of ITRs, especially when compared to standard school-based sealant programs. As such, it is not clear if the effects of primary and/or secondary prevention are consistently linear as children age. GAMs are therefore useful in the analysis of longitudinal caries data because of their flexibility in modeling nonlinear effects. GAMs were used to estimate smoothed coefficients for treatment effects over time. To obtain parametric estimates for covariates included as smoothed terms in additive models, identical models were run using generalized estimating equations (untreated decay) and multilevel Poisson regression (TOCE).

Decay prevalence was modeled as a binomially distributed variable, *Y*_*i*_~*Bin*(*π*_*i*_), using a logit link function, and TOCE scores were modeled using a Poisson family with a log link function. Models for both outcomes included smoothed terms, specified for untreated decay as $logit\left({\pi}_i\right)=\ln \left(\frac{\pi_i}{1-{\pi}_i}\right)=\theta \boldsymbol{T}+{s}_1\left({x}_{1i},\ast z\right)+{s}_2\left({x}_{2i}\right)+\beta (z)$, where *π*_*i*_ is the probability of decay, *x*_*i*_ is time, **T** is a vector of linear covariates and *θ* their corresponding beta coefficients, *s*_1_ is a smoothing term for the number of preventive treatments received, and *s*_2_ is a smoothed term for the participants’ age at the time of examination. Effectiveness of prevention with ITRs versus sealants only was assessed using a dummy variable for exposure group (*z*) and a nonlinear treatment/time interaction. Smoothing terms were estimated using cubic regression splines. Covariates included baseline TOCE scores, gender, whether the participant had access to fluoridated water, and receipt of previous dental care, determined by children presenting with new fillings or restorations at any observation that were not placed in previous preventive treatments.

For each outcome in GAM analyses, a series of models were specified that included no smoothing terms, smoothers for time, smoothers for age at examination, and smoothers for the treatment/time interaction. The upper limit for degrees of freedom associated with smoothed terms was set at five. The degree of nonlinearity for smoothed coefficients was assessed using the effective degrees of freedom (edf), with greater departures from linearity evidenced by edf coefficients being different from one. The comparative fit of nested additive models was evaluated using deviance scores and compared to a χ^2^ distribution with *df* equal to the number of additional parameters in the non-nested model. Plots were produced for nonlinear trends within each treatment group for TOCE.

Analysis was conducted in R v3.4.2. Statistical significance was set at 0.05. This study received IRB approval from the Forsyth Institute and the NYU School of Medicine.

## Results

The analytic sample (*n* = 8207) consisted of 6584 participants in the cohort receiving primary and secondary prevention and 1623 participants in the primary prevention only group (Table 1). The average age of participants at baseline was equivalent across exposed (7.32 ±1.71) and unexposed (7.39 ±1.93) groups. Approximately 51% of participants were male, and 60% had evidence of receiving previous dental care. Overall, 32.7% of participants had untreated decay on any dentition at baseline, and 9.2% had untreated decay on any permanent tooth. Baseline untreated decay on permanent teeth was similar across exposed (9.3%) and unexposed (8.7%) groups. However, the average total observed caries experience at baseline was larger in the exposed group (2.19 ±2.83) compared to those unexposed (1.55 ±2.13). Thus, multivariable models further adjusted for baseline TOCE scores. Changes in TOCE and untreated decay by visit are shown in Table 2.

**Table 1:** Baseline sample descriptive statistics

	All (*n* = 8207)		Exposed (*n* = 6584)		Unexposed (*n* = 1623)	
	mean	SD	mean	SD	mean	SD
Age at exam	7.33	1.76	7.32	1.71	7.39	1.93
TOCE	2.06	2.72	2.19	2.83	1.55	2.13
	*N*	%	*N*	%	*N*	%
Males	3840	50.87	3338	51.4	502	47.58
Previous dental care	4942	60.22	3986	60.54	956	58.9
Decay at baseline (any)	2683	32.69	2211	33.58	472	29.08
Decay at baseline (permanent teeth)	755	9.20	614	9.33	141	8.69

Notes: Exposed group received primary and secondary prevention, unexposed received primary prevention only. *TOCE* total observed caries experience

**Table 2:** Average TOCE and prevalence of untreated decay on permanent teeth, by visit

	All (*n* = 8207)		Exposed (*n* = 6584)		Unexposed (*n* = 1623)	
	mean	SD	mean	SD	mean	SD
TOCE						
Baseline	2.06	2.72	2.19	2.83	1.55	2.13
Visit 1	2.68	3.18	2.82	3.31	2.04	2.44
Visit 2	3.32	3.58	3.55	3.72	2.28	2.63
Visit 3	3.66	3.71	3.88	3.84	2.58	2.75
Visit 4	3.98	3.74	4.27	3.87	2.77	2.73
Visit 5	4.27	3.81	4.60	3.91	2.72	2.82
Visit 6	4.81	3.87	5.44	3.97	2.98	2.93
	*N*	%	*N*	%	*N*	%
Untreated decay						
Baseline	755	9.20	614	9.33	141	8.69
Visit 1	457	7.31	379	7.42	78	6.84
Visit 2	280	8.82	226	8.72	54	9.29
Visit 3	152	7.13	127	7.15	25	7.00
Visit 4	72	6.90	52	6.16	20	10.00
Visit 5	18	3.24	16	3.49	2	2.06
Visit 6	11	6.01	7	5.15	4	8.51

Note: *TOCE* total observed caries experience

Model results for GEE and multilevel analyses (Table 3a) indicate that the risk of untreated decay in permanent teeth significantly decreased over time across both cohorts (OR = 0.77, 95% CI = 0.72, 0.82), and that risks were significantly lower in the exposed group compared to unexposed (OR = 0.77, 95% CI = 0.60, 0.98). Increases in baseline total observed caries experience were significantly associated with higher risk of decay (OR = 1.12, 95% CI = 1.09, 1.15). Gender, previous dental care, and water fluoridation were not statistically associated with untreated decay. The interaction between treatment and time was not significant and thus was not included in the final model.

**Table 3:** Model results comparing caries prevention programs over time

	TOCE				Untreated decay (permanent teeth)			
3a: Coefficients (GEE and multilevel models)	IRR	*p*	95% CI		OR	*p*	95% CI	
Visit (trend)	1.20	< .001	1.18	1.23	0.77	< .001	0.72	0.82
Exposed (vs unexposed)	0.95	0.274	0.88	1.04	0.77	0.034	0.60	0.98
Baseline TOCE	1.45	< .001	1.43	1.46	1.12	< .001	1.09	1.15
Age at exam	1.01	0.038	1.00	1.03	1.27	< .001	1.21	1.33
Sex (Male)	0.99	0.517	0.95	1.03	1.06	0.468	0.90	1.25
Previous dental care	1.42	< .001	1.35	1.49	0.93	0.457	0.78	1.12
Water fluoridation	1.15	< .001	1.09	1.20	1.07	0.486	0.88	1.29
Treatment*Visit	0.96	0.001	0.94	0.98	–	–	–	–
3b: Coefficients (smoothed GAM)^a^	IRR	p	95% CI		OR	*p*	95% CI	
Baseline TOCE	1.26	< .001	1.25	1.26	1.15	< .001	1.12	1.19
Sex (Male)	0.99	0.330	0.98	1.01	1.09	0.980	0.92	1.23
Previous dental care	1.43	< .001	1.40	1.45	0.79	0.020	0.65	0.96
Water fluoridation	1.12	< .001	1.11	1.14	0.94	0.510	0.79	1.13

^a^Notes: Exposed group includes participants receiving primary and secondary prevention. Unexposed group includes participants receiving primary prevention only. Parametric effects for age, exposure (treatment), and visit (non-smoothed) are shown for standard GEE (adult decay) and multilevel (TOCE) models for comparison purposes (model 3a). Age, visit, exposure (treatment), and the visit-treatment interaction were included in model 3b as smoothed terms in GAMs and do not have estimated parametric coefficients. Nonlinear effects for these terms in the generalized additive model are shown in Table 4

*TOCE* total observed caries experience, *GEE* generalized estimating equations, *GAM* generalized additive models, *IRR* incident rate ratio, *OR* odds ratio

The total number of observed decayed or filled teeth (TOCE) was not significantly different across exposure groups (IRR = 0.95, 95% CI = 0.88, 1.04) in adjusted models (Table 3a), and linear trends in TOCE indicated a significant increase in total observed caries over time (IRR = 1.20, 95% CI = 1.19, 1.23). However, the interaction between treatment and time was statistically significant (IRR = 0.96, 95% CI = 0.94, 0.98), indicating that the rate of TOCE increase slowed over time for participants receiving primary and secondary prevention compared to participants receiving primary prevention alone. While no differences in TOCE were found by gender, previous dental care and water fluoridation were both associated with an increased rate of new decayed or filled teeth. The fully adjusted model for TOCE accounted for 63% of the deviance.

Estimates for covariates not included as smoothed parameters in generalized additive models were similar in magnitude and significance to those from GEE and multilevel models (Table 3b).

Nonlinear effects (Table 4) for TOCE over time as measured by the effective degrees of freedom from additive models were 3.46 and 2.30 for exposed and unexposed groups, respectively. For untreated decay, effects were 1.12 and 1.00 for exposed and unexposed groups, close to a linear trend. Nonlinear coefficients by group were all statistically significant despite minimal departures from linearity for untreated decay.

**Table 4:** Nonlinear effects for age at examination and trends by exposure group estimated by smoothed terms

	TOCE		Untreated decay (permanent teeth)	
Smoothing Terms (GAMs)	edf	*p*	edf	*p*
Age at exam	2.94	< .001	3.85	< .001
Visit among unexposed	2.30	< .001	1.00	0.02
Visit among exposed	3.46	< .001	1.12	< .001

Note: increased magnitude of effective degrees of freedom (edf) indicates greater departures from linearity

*TOCE* total observed caries experience, *GAM* generalized additive models

**Fig. 1:**
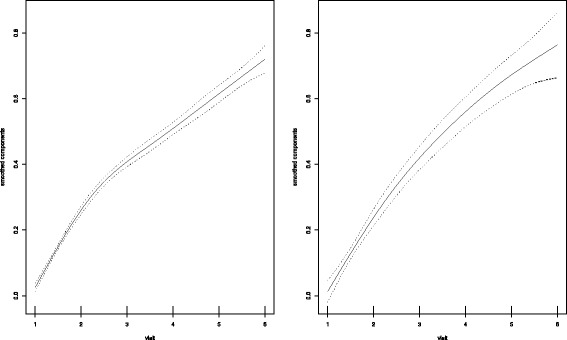
Smoothed trends in TOCE over time for exposed (left) and unexposed (right) groups

Plots for smoothed coefficients over time for TOCE by exposure as estimated by additive models are shown in Fig. 1. Trends in both groups as shown in Fig. 1 indicate an increase in estimates for TOCE over time.

## Discussion

The large scale implementation of school-based caries prevention can substantially increase dental care utilization for high-risk children, particularly those from low-SES backgrounds or those living in medically underserved areas [12]. However, designing a program with the optimum mix of treatments and frequency of care requires additional research. While the interventions commonly used in school-based prevention are efficacious in clinical trials for preventing caries, evidence of effectiveness in community-based settings is largely limited to sealants or fluoride or is restricted in follow-up time [6, 13–17]. In this study, we sought to compare primary versus secondary prevention agents and explore trends in decay risk in primary school-aged children. Overall, school-based caries prevention across programs was associated with a reduced risk of untreated decay in permanent teeth over time, and a prevention program with higher treatment intensity was associated with a significantly lower risk of untreated decay. Specifically, children receiving prevention that included interim therapeutic restorations and sealants on all teeth had a lower risk of untreated decay in permanent dentition compared to prevention with sealants placed on only the first and second molars. While TOCE increased over time across groups, the rate of new carious teeth over time was lower for participants receiving primary and secondary prevention. Nonlinear trends over time by group were also statistically significant.

This study included a large sample of US primary-school aged children (aged 5–12 years) from the northeastern and central United States and analysis controlled for baseline TOCE scores, gender, evidence of previous dental care, and water fluoridation. Though data were not available on race/ethnicity and socioeconomic status, all participants were enrolled in Title-1 schools, determined by having a large majority of enrolled students receiving free and reduced-price lunch. Additionally, there were no differences between groups in baseline untreated decay. Thus, it is not believed that observed treatment effects are due to imbalances in SES or other unobserved covariates. Further, a key feature of school-based caries prevention is the large-scale effectiveness for high-risk children with limited access to dental care. For study participants in both cohorts, dental access outside of the study was minimal. The findings from this study may be generalizable to other high-risk, low-income primary school populations.

In this study, there were no differences in overall TOCE comparing children receiving sealants and ITRs versus sealants alone. This finding is similar to the existing ITR literature. For example, a five-year study of early childhood caries in India showed that the use of ITRs in combination with fluoride varnish and sealants did not significantly decrease the incidence of new caries [18]. However, this prior study also found no impact on the prevalence of untreated decay, which is in contrast to the presented results. Notably, as interim therapeutic restorations are considered secondary prevention, they are only placed on erupted carious lesions. Thus, cavitated teeth or tooth surfaces treated with ITRs should show a potential decrease in untreated decay over time, but would still contribute to overall TOCE scores.

While results show an overall significant increasing trend in TOCE over time across groups, the rate was significantly lower in the exposure group compared to those unexposed. Interim restorations have been previously shown to reduce the levels of cariogenic oral bacteria, potentially preventing the progression of decay and lowering the rate of additional caries on adjacent teeth due to increases in unchecked bacteria [19]. Thus, the survival rate of ITRs may reduce untreated decay in school-aged children, particularly those without regular access to dental care, and help in preventing new caries. Although the decreased rate of TOCE in those receiving primary and secondary prevention was small (as evidenced by both model results and the similarities in trends over time in associated figures), TOCE scores were calculated using data from all teeth and tooth surfaces. ITRs may be even more effective at reducing the subsequent risk of decay on teeth that are adjacent to the tooth that was recently restored. Future research should isolate teeth that are proximal to those with applied ITRs to investigate this hypothesis further.

Nonlinear effects for TOCE indicated a cubic trend for prevention that included sealants and ITRs, and a quadratic trend for prevention consisting of sealants only. Effects for untreated decay indicated an approximately linear trend for both groups. However, despite the significant nonlinear effects, plots of TOCE by group over time suggest that a linear trend in dental caries may be sufficient for program planning over the short or medium-term. Importantly, the significance of smoothed terms was determined using χ^2^ tests. Thus, the large sample size in this study could result in significant differences for even small departures from linearity. For the long-term effects of caries prevention, trends over time are expected to be nonlinear.

This study has a number of limitations. Despite the use of longitudinal data, analysis was limited by diminishing sample sizes at later observations and the number of available observational periods. It has been shown that estimation of smoothed terms is difficult with longitudinal data that is characterized by limited observations [20]. Though this can be simplified by using fewer knots, this can lead to biased estimates [20]. Collecting more data, either by extending the length of follow-up or increasing the number of observations between treatments, could result in more robust estimation of trends over time. This would also support the use of generalized additive mixed models, adding potential random effects for trend, treatments, and individual subjects to explore treatment effects in even greater detail [21].

While this study was open to all students with completed informed consent, the overall participation rate was approximately 30%. Thus, study participants may have different oral health needs than nonparticipants. As study interventions were provided at the school level, children who move, graduate, change schools, or are otherwise lost to follow-up did not receive continued care. As a result, true effects over extended time periods are unknown. Further, though estimates of exposures are believed to be robust, there may be residual confounding bias due to missing student-level socioeconomic status and race/ethnicity. This is perhaps best demonstrated by the significant increase in TOCE for children with access to water fluoridation, which was unexpected. Finally, children in both cohorts were examined and treated biannually, resulting in interval censoring between visits. While both cavitated and filled teeth were included in analysis, any tooth that may have developed decay and was subsequently lost in intervening periods would be unobserved.

## Conclusions

This study evaluated the effectiveness of ITR and sealants on untreated caries and the total observed caries experience, compared to sealants only. Results showed that ITR plus sealants was associated with a decrease in untreated dental caries in permanent dentition and a slightly lower rate of total caries increase over time. Despite significant nonlinear coefficients across groups over time, visual inspection of plots of smoothed coefficients suggest a linear approximation for short or long-term prevention studies may be appropriate. Given the high variability in school-based caries prevention, comparing the effectiveness of different prevention agents, frequency, or intensity of care can lead to optimal program design.
